# Early-stage multi-cancer detection using an extracellular vesicle protein-based blood test

**DOI:** 10.1038/s43856-022-00088-6

**Published:** 2022-03-17

**Authors:** Juan Pablo Hinestrosa, Razelle Kurzrock, Jean M. Lewis, Nicholas J. Schork, Gregor Schroeder, Ashish M. Kamat, Andrew M. Lowy, Ramez N. Eskander, Orlando Perrera, David Searson, Kiarash Rastegar, Jake R. Hughes, Victor Ortiz, Iryna Clark, Heath I. Balcer, Larry Arakelyan, Robert Turner, Paul R. Billings, Mark J. Adler, Scott M. Lippman, Rajaram Krishnan

**Affiliations:** 1 Biological Dynamics, Inc, San Diego, CA USA; 2 WIN Consortium for Personalized Cancer Therapy, Villejuif, France; 3 Medical College of Wisconsin, Milwaukee, WI USA; 4 University of Nebraska, Omaha, NE USA; 5 The Translational Genomics Research Institute (TGen), Phoenix, AZ USA; 6 City of Hope National Medical Center, Duarte, CA USA; 7 University of California San Diego, Departments of Family Medicine and Public Health, San Diego, CA USA; 8 University of Texas MD Anderson Cancer Center, Department of Urology, Houston, TX USA; 9 University of California San Diego, Moores Cancer Center, San Diego, CA USA; 10 San Diego Cancer Research Institute, San Diego, CA USA

**Keywords:** Bladder cancer, Cancer screening, Ovarian cancer, Pancreatic cancer

## Abstract

**Background:**

Detecting cancer at early stages significantly increases patient survival rates. Because lethal solid tumors often produce few symptoms before progressing to advanced, metastatic disease, diagnosis frequently occurs when surgical resection is no longer curative. One promising approach to detect early-stage, curable cancers uses biomarkers present in circulating extracellular vesicles (EVs). To explore the feasibility of this approach, we developed an EV-based blood biomarker classifier from EV protein profiles to detect stages I and II pancreatic, ovarian, and bladder cancer.

**Methods:**

Utilizing an alternating current electrokinetics (ACE) platform to purify EVs from plasma, we use multi-marker EV-protein measurements to develop a machine learning algorithm that can discriminate cancer cases from controls. The ACE isolation method requires small sample volumes, and the streamlined process permits integration into high-throughput workflows.

**Results:**

In this case-control pilot study, comparison of 139 pathologically confirmed stage I and II cancer cases representing pancreatic, ovarian, or bladder patients against 184 control subjects yields an area under the curve (AUC) of 0.95 (95% CI: 0.92 to 0.97), with sensitivity of 71.2% (95% CI: 63.2 to 78.1) at 99.5% (97.0 to 99.9) specificity. Sensitivity is similar at both early stages [stage I: 70.5% (60.2 to 79.0) and stage II: 72.5% (59.1 to 82.9)]. Detection of stage I cancer reaches 95.5% in pancreatic, 74.4% in ovarian (73.1% in Stage IA) and 43.8% in bladder cancer.

**Conclusions:**

This work demonstrates that an EV-based, multi-cancer test has potential clinical value for early cancer detection and warrants future expanded studies involving prospective cohorts with multi-year follow-up.

## Plain Language Summary

Finding cancer early can make treatment easier and improve odds of survival. However, many tumors go unnoticed until they have grown large enough to cause symptoms. While scans can detect tumors earlier, routine full-body imaging is impractical for population screening. New cancer detection methods being explored are based on observations that tumors release tiny particles called extracellular vesicles (EVs) into the bloodstream, containing proteins from the tumor. Here, we used a method to purify EVs from patients’ blood followed by a method to detect tumor proteins in the EVs. Our method quickly and accurately detected early-stage pancreatic, ovarian, or bladder cancer. With further testing, this method may provide a useful screening tool for clinicians to detect cancers at an earlier stage.

## Abstract (web summary)

Hinestrosa et al. describe the early-stage detection of cancer using biomarkers present in circulating extracellular vesicles purified via an alternating current electrokinetics platform. They show, in a case-control study, that 95.7% of pancreatic, 75.0% of ovarian and 43.8% of bladder stage I and II cancers can be detected.

## Introduction

Detecting cancer early before symptoms present is key to improving patient survival. While not all emergent tumors will become deadly, for those destined to become so, the ability to treat the disease while it is still localized is a major factor for improving 5-year survival rates^1^. Pancreatic ductal adenocarcinoma (PDAC), one of the deadliest cancers and a leading cause of all cancer-related deaths in the United States, typically goes undetected until it spreads and becomes unresectable and metastatic^2^. In contrast, for the few patients (~15%) diagnosed with localized disease, the 5-year survival rate rises to about 25% and when PDAC is detected at Stage I, survival rates can be as high as 80%^3^. Likewise, ovarian cancer typically has few symptoms and is often undetected until it is advanced, with 5-year survival rates of <31%^4^. When detected early, 5-year survival rates for localized ovarian cancer jump to a remarkable 93%, but currently, only 15% of cases are detected at early stages^5^. For metastatic bladder cancer, 5-year survival rates are only 6%, while detection when the tumor is still localized to the bladder wall inner layer results in an improved 5-year survival rate of 96%^6^. Importantly, treatment of localized bladder cancer has less morbidity and better quality of life compared to treatments required for metastatic cancer^7^.

Currently, there are few general screening strategies to detect asymptomatic, early-stage PDAC, ovarian, or bladder cancer^8^. Given recent advances in targeted treatments for cancer, which are based on functional changes in the genome and proteome of individual tumors and their milieu, attention has turned to the possibility of detecting these changes directly from blood, i.e., by liquid biopsy^1,9^. This strategy has been utilized to design multi-cancer early detection (MCED) tests that involve blood-based circulating proteins and/or DNA mutations and methylations followed by machine learning approaches to discern between cancer and non-cancer cases^10–15^. Several MCED tests based on these approaches are being developed and have shown promise for detecting clinically significant, late-stage (III and IV) cancers, and that detection was prognostic beyond tumor stage^16^. Detecting early, stage I, and II cancers with high enough sensitivity for population-level screening, however, has proven much more challenging^12–14^.

One potential approach for more sensitive detection of cancer-related biomarkers from blood involves the use of extracellular vesicles (EVs) such as exosomes, 30 to 150 nm vesicles that mediate cell-to-cell communication^17,18^. It has been shown that some exosomes are ejected by tumors into the bloodstream and they carry functional protein biomarkers representing the tumor proteome^18,19^. The potential to better detect cancer using EV-bound protein biomarkers has recently been suggested for multiple cancer types^20^ using various methodologies, such as mass spectrometry for lung and pancreatic cancers^21^. Currently, the gold standard method for isolating EVs from soluble contaminants (cells, small proteins, or other vesicles) is ultracentrifugation (UC), which is inefficient and not suitable for point-of-care applications^22^. To address this issue, many groups have explored a variety of methods, based on immunoaffinities and/or diverse membranes, to both isolate and analyze circulating EVs and associated markers^17^. In this study, we used an alternating current electrokinetic (ACE)-based platform (Verita™ System)^23^ to efficiently purify exosomes and other EVs from patient samples, then measured the concentrations of associated protein biomarkers (“EV proteins”) present in the purified EV samples from our case-control study subjects. Using the information about the differing EV protein concentrations, we developed a machine-learning algorithm to identify a small set of EV biomarkers which together with age permits detection of early-stage pancreatic, ovarian, and bladder cancers. We find that using ACE purification of EVs, followed by a specialized analysis of the EV protein biomarkers, successfully predicts the presence of early stage I–II pancreatic, ovarian, and bladder cancers with a sensitivity of 71.2% (95% CI: 63.2–78.1) at 99.5% (97.0–99.9) specificity, and an AUC of 0.95 (95% CI: 0.92–0.97). To our knowledge, we are the first to report feasibility for a blood based, MCED test for the detection of stage I and II cancers that employs circulating EV proteins exclusively.

## Methods

### Sample collection and processing

All specimens for this retrospective study were collected over a period of several years by a commercial biorepository (ProteoGenex, Inglewood, CA, USA). Stage I and II samples were selectively obtained from available inventory. Samples had been collected from patients in hospital settings and following collection were maintained by the commercial biorepository. All relevant ethical regulations were followed, and informed consent was obtained prior to sample collection. The protocol was approved by the ethics committee at the N. N. Blokhin National Medical Research Center of Oncology. In the hospital settings, potential cancer patients were identified by any suspicious findings arising during imaging that was conducted either in response to patient symptoms or as part of routine, annual examinations. We do not have access to information on which patients were symptomatic and which were asymptomatic. Cancers were confirmed via subsequent tissue biopsy and staged by pathologists in the hospital using pathology and surgical reports, according to AJCC (7th edition) guidelines, along with imaging to assess any spread to distant sites. All subjects with confirmed diagnosis of cancer were treatment naive (prior to surgery, local, and/or systemic anti-cancer therapy) at the time of blood collection. The biorepository provided the patient samples along with demographics, surgical, and pathology information. Through the analysis of these data, staging for patients was reviewed a second time for accuracy by the study authors. Our study did not require ethics approval because samples were de-identified after processing by the biorepository. Since ovarian cancer patients did not uniformly undergo comprehensive surgical staging, an occult disease higher than the indicated stage cannot be ruled out. The control group has no known history of cancer, autoimmune diseases, or neurodegenerative disorders, nor any presence of diabetes mellitus (types 1 and 2). A total of 323 subjects were included in the study, including 139 subjects (‘Cancer case patient cohort’) who were diagnosed with one of the three cancers between January 2014 and September 2020. In the cancer case cohort, whole venous blood specimens were collected shortly before biopsy (median −1 day, mean −2.7 days), and prior to surgical intervention, radiation therapy, or cancer-related systemic therapy. The median age was 60 years [Min–Max 21–76] in the cancer case cohort (*N* = 139, 56 males, 83 females) and 57 years [Min–Max 40–71] in the control cohort (*N* = 184, 82 males and 82 females). Details on the case-control cohorts can be found in Supplementary Data 1 and Supplementary Data 2. Whole blood samples were collected in K_2_EDTA plasma vacutainer tubes and processed into plasma within 4 h of collection. The whole blood was first spun at 1500 × *g* for 10 min at 4 °C with no brake used. After the first spin, plasma was transferred into fresh tubes and subjected to a second spin at 1500 × *g* for 10 min. After the second spin, plasma was aliquoted into 1 mL tubes and frozen within 1 h at −80 °C. All specimens used in this study were processed under identical conditions.

### EV/exosome isolation and particle characterization

#### Isolation of EVs using the Verita™ Platform

**Fig. 1:**
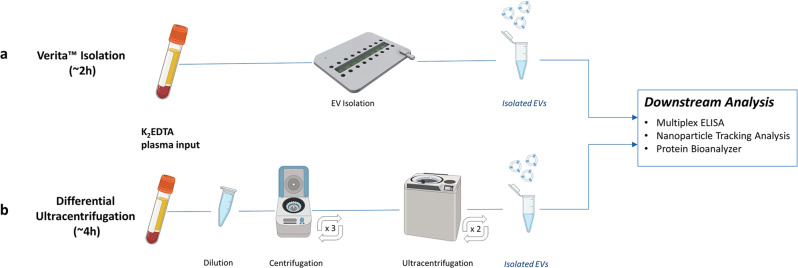
Schematic showing EV isolation workflows using either Verita™ or ultracentrifugation methods. **a** Workflow using the Verita™ Isolation platform. As plasma samples are flowed onto the energized AC Electrokinetics (ACE) microelectrode array, EVs are collected onto the electrodes. Unbound materials are removed with a buffer wash, the electric field turned off, and EVs are eluted into the buffer. **b** Workflow for differential ultracentrifugation. Plasma samples are diluted, and large debris pelleted by a low-speed centrifugation step. Supernatants are removed and subjected to two additional cycles of low-speed centrifugation. EVs in the cleared supernatants are then ultracentrifuged two times, and lastly the pellet is resuspended in buffer.

EVs, including exosomes, were extracted from plasma as previously described using an AC Electrokinetic (ACE)-based isolation method (Biological Dynamics, CA, USA) as shown in Fig. 1^23–25^. Briefly, 240 µL of each undiluted plasma was introduced into a Verita™ chip, and an electrical signal of 7 Vpp and 14 KHz was applied while flowing the plasma across the chip at 3 µL/min for 120 min. EVs were captured onto the energized microelectrode array, and unbound materials were washed off the chip with Elution Buffer I (Biological Dynamics) for 30 min at 3 µL/min. The electrical signal was turned off, releasing EVs into the solution remaining on the chip (35 µL), which was then collected, and this solution containing purified, concentrated/eluted EVs was used directly for further analysis. This method has also been used previously for the isolation of cell-free DNA, exosomal RNA, and exosomal protein markers in both solid-tumors and hematological malignancies^24–29^. The Verita-purified EVs were characterized using nanoparticle tracking analysis (NTA) via ZetaView instrument (Particle Metrix, Inning am Ammersee, Germany). Supplementary Data 3 shows the particle size and concentration values for the EVs isolated from each subject in the cohorts while Fig. S1 shows comparisons between the case and control cohorts.

#### Isolation of EVs via differential ultracentrifugation

A subset of case and control samples were subjected to differential ultracentrifugation as a conventional means of EV isolation^21^. In brief, 760 µL of 1× PBS was added to 240 µL of each subject plasma, then spun successively at 500 × *g* for 10 min, 3000 × *g* for 20 min, and 12,000 × *g* for 20 min, collecting the supernatants after each step. Subsequently, the resulting supernatant was subjected to ultracentrifugation at 100,000 × *g* for 70 min, pellets were washed in 1× PBS and then ultracentrifuged again at 100,000 × *g* for 70 min. The supernatant was discarded, and the resulting pellet was resuspended in 120 µL of 1× PBS for further analysis (Fig. 1).

#### Protein contamination analysis

To determine the presence of contaminating total protein in the EV preparations from both the Verita™ platform and the differential ultracentrifugation process, samples were analyzed using the Qubit 4 fluorometer (ThermoFisher Scientific, Waltham, MA) with the Qubit™ Protein quantitation assay (Cat No. Q33212, ThermoFisher Scientific, Waltham, MA), run according to manufacturer specifications. To further understand the composition of the contaminating proteins on the isolation products, we used the 2100 Bioanalyzer (Agilent, Santa Clara, CA) with the Protein 230 kit for protein analysis (Cat No. 5067-1517) following the manufacturer’s directions.

#### Protein biomarker analysis

Verita-isolated EV samples, as well as original, unpurified plasma samples from the same patients, were used directly in commercial multiplex immunoassays to quantify the presence of marker proteins. In brief, 25 µL of each purified EV sample was used for analysis by each of three different bead-based immunoassay kits, according to the manufacturer’s directions for each kit (Human Circulating Biomarker Magnetic Bead Panel 1 (Cat # HCCBP1MAG-58K), Human Angiogenesis Magnetic Bead Panel 2 (Cat # HANG2MAG-12K), and Human Circulating Cancer Biomarker Panel 3 (Cat # HCCBP3MAG-58K); Millipore Sigma, Burlington, MA). Protein biomarker concentration was assessed using the MAGPIX system (Luminex Corp, Austin, TX) according to the manufacturer’s protocols. Belysa software v. 3.0 (EMD Millipore) was used to determine final protein concentrations from the calibration curves. Limit of detection (LOD) and units of measure for each of the biomarkers are listed in Supplementary Data 4.

#### Spike EV isolation models for EV biomarker signal

To further understand the presence of relevant protein biomarkers on the EVs, we employed EVs purified from cell culture supernatants representing two different cell lines as positive controls. The cell line H1975 (ATCC CRL-5908™) is known to express the CA19-9 marker (ST6 N-acetylgalactosaminide alpha-2,6-sialyltransferase 1) while the cell line HeLa (ATCC CRM-CCL-2™) is known to express the CA 125 marker (Mucin16). Briefly, the H1975 EVs were spiked at three different concentrations (from 4.60 × 10^9^ to 1.15 × 10^9^ particles/mL) into K_2_EDTA plasma, the EVs were isolated using the Verita™ platform, and subsequently analyzed via a bead-based immunoassay for the presence of the CA 19-9 biomarker. The linear response to EV input (marker values ± standard deviation) is shown in Fig. S2. In another experiment, the H1975 EVs and the HeLa EVs were spiked into K_2_EDTA plasma and isolated using the Verita^TM^ platform. The biomarker reading results confirm the positive detection of the respective expected signals with CA19-9 being elevated for the H1975 EVs and CA 125 being elevated for the HeLa EVs (Fig. S2).

### Statistics and reproducibility

Each case or control sample was measured in duplicate or triplicate (depending on volume availability) with one chip eluate going to one reaction well in the multiplex immunoassay plate. No pooling or dilution of the eluates was performed. The same approach was followed for the differential ultracentrifugation experiments where each immunoassay well contained a single resuspended pellet.

#### Biomarker selection

**Fig. 2:**
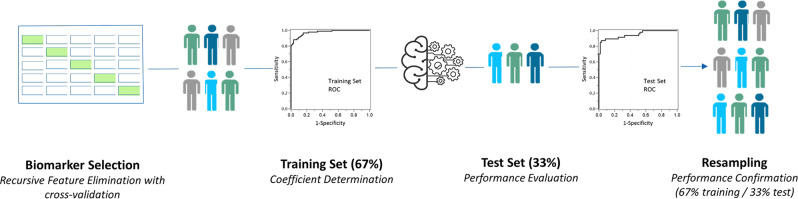
Development of classification algorithm for multi-cancer early detection. Biomarker selection is performed via recursive feature elimination (RFE) with cross validation. After the biomarkers are selected, the dataset is split into training and test sets. The training set is used for the determination of the coefficients in the logistic regression for each biomarker and the test set is used to evaluate the performance of the logistic regression fit from the training set in a held-out test set. Finally, the process of splitting the dataset into training and test sets is randomly repeated 100 times for performance confirmation.

From an initial evaluation of 42 EV proteins, 34 different biomarkers with <50% of values missing or below the LOD were considered (Supplementary Data 2 a**nd** Supplementary Data 4). In cases with missing values or results below the LOD, values were set (imputed) to the LOD. Distributions for all biomarkers were evaluated and distributions were found to be wide; thus, we used a Log2 transformation on all EV protein biomarker values in subsequent analyses. Subsequently, we explored the correlations among the biomarkers using the R module ‘Corrplot’ to determine the potential for multicollinearity in building classification models (correlation heatmap from all the biomarkers measured are shown in Fig. S3). To determine the most informative biomarkers, recursive feature elimination with fivefold cross-validation^30^ was employed using the R module “caret”. In this methodology, four of the five folds are used for selecting a subset of biomarkers using stepwise backwards selection. This process is repeated five times, using each fold once as a held-out test set. As the folds of cross-validation are chosen at random, this was repeated 100 times and the subset of biomarkers that maximized the partial AUC (pAUC)^31^ over the range of specificities from 0.75 to 1.00 across all test sets was selected (Fig. 2).

### Coefficient determination and performance evaluation

Once the biomarkers were selected, an initial partition of the data into training (67%) and test (33%) sets, stratified by cancer types, allowed us to determine the performance of the biomarkers selected by estimating the regression coefficients for the model using the training set and evaluating the classification performance in the held-out test set (Fig. 2). To pursue a fair assessment of our model, given our relatively small sample size and to avoid overfitting^30,32–34^, we resampled 100 independent training and test sets (made up of 2/3 and 1/3 of the 323 individuals stratified by cancer type) from the overall data set. The subjects in the training set, for each resample, were used to estimate biomarker regression coefficients in the model whereas the diagnostic performance was assessed independently in subjects in the held-out test set. Receiver-operator characteristic (ROC) curves, area under the curve (AUC), sensitivity, specificity, and related metrics were computed for the test sets based on the individual fits for each of the subjects in each respective partition (Supplementary Data 5). For each of the held-out test sets, a threshold determination of >99% specificity was computed (because there were 61 control subjects in each held-out test set, this effectively means calling 61 out of 61 correctly) and subsequently, the average threshold was determined (Supplementary Data 6). Using the average threshold and the average fit in the test set for each subject, we then evaluated the performance for the overall cohort as well as for subcohorts (e.g., pancreatic cancer). The 95% confidence intervals for AUC were calculated using a bias-corrected bootstrapping method (*N* = 2000)^35^ while the confidence intervals for performance metrics, i.e. sensitivity and specificity, were calculated based on the Wilson two-sided method^36^. During the evaluation of the logistic regression model, we assessed the importance of each biomarker selected using the average standardized coefficients (Supplementary Data 7). Here “importance” can be understood as a quantitative comparison between predictors. One predictor is more important than another if it contributes more to the prediction of the response variable across all the models considered in the regression.

#### Additional analysis and plotting

Additional analysis and plotting in both the main text and the supplementary information were performed in GraphPad Prism (Version 9.0.2) and JMP Pro (Version 16.1.0).

### Reporting summary

Further information on research design is available in the Nature Research Reporting Summary linked to this article.

## Results and discussion

Using the Verita™ system, we isolated EVs from both control plasma and plasma from stage I and II pancreatic, ovarian, and bladder cancer cases (Fig. 1, Supplementary Data 1 and Supplementary Data 2). Previous studies have shown that the EV populations isolated using the ACE technology are consistent with the presence of exosomes, in accordance with the ISEV 2018 guidelines^37^ (mean particle sizing ~120 nm; CD63-positive; TSG101 can be detected only following membrane permeabilization; SEM images display rounded, cup-shaped morphology; contain functional RNA)^23,24,28^. After EV isolation, we measured the particle size distribution and concentration and confirmed equivalent isolation for both cohorts (Supplementary Data 3 and Fig. S1). To simulate a real-world screening scenario, all cancer cases were treatment-naïve; to ensure that these were early-stage patients, the histopathologic staging was confirmed using the American Joint Commission on Cancer (AJCC) guidelines. The median age of the cancer cases was 60 years (59.7% female, 40.3% male; Supplementary Data 1). Notably, 63.3% of the overall cancer cases were stage I, with the remaining 36.7% at stage II. Furthermore, the stage I ovarian cohort was comprised predominantly (60%) of stage IA samples. The control group had no known history of cancer, autoimmune diseases, or neurodegenerative disorders, nor any presence of diabetes mellitus, with a median age of 57 years (50.0% female, 50.0% male).

**Fig. 3:**
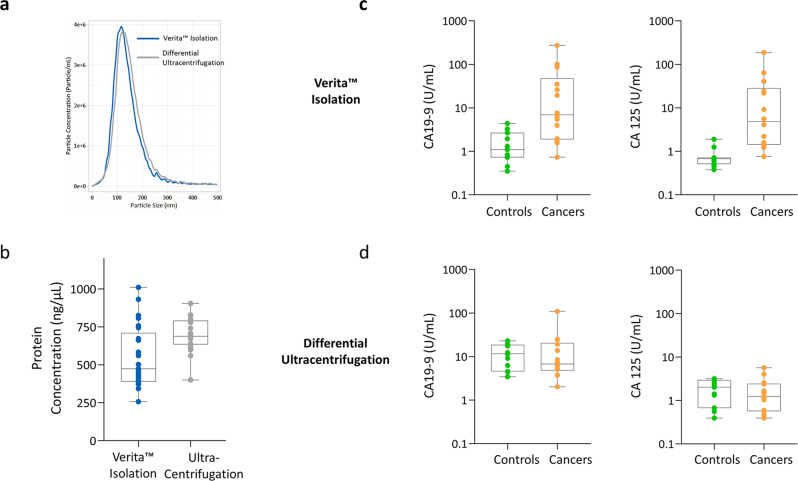
Characterization of EVs isolated by either Verita™ or differential ultracentrifugation. **a** Distribution of particle sizes as determined by nanoparticle tracking analysis. Blue line represents the particle distribution from Verita™ isolation (*N* = 25 subjects) while the gray line represents the isolation from differential ultracentrifugation (*N* = 25 subjects). **b** Levels of residual contaminating total proteins based on Qubit™ protein assay (*N* = 25 subjects for each isolation methodology). The ability to differentiate cancer cases from controls based on biomarkers CA 19-9 and CA125 is shown for EVs isolated using the Verita™ isolation in panel (**c**), and EVs isolated by differential ultracentrifugation in panel (**d**). In both (**c**), (**d**) panels, the N for Controls is 11 subjects, and the *N* for Cancers is 14 subjects.

To evaluate the advantages of using ACE-isolated EVs for proteomic analysis, EVs were isolated from a subset of case and control patient samples using either Verita™ or a differential ultracentrifugation method (Fig. 1). Following isolation, the only physical difference observed between the two methods was a slight decrease in average particle size for EVs isolated on the Verita™ platform (138 nm for UC versus 120 nm for ACE EVs; Fig. 3a). Further breakdown of the particle size distributions is shown in Fig. S4. When EVs prepared by the two methods were assessed for total plasma protein content, the UC EV preparations were found to contain much higher levels than the ACE EVs (Fig. 3b). For example, contamination with the plasma protein IgG was much higher in the UC isolated material (Fig. S4). This is consistent with previous reports that UC-prepared EVs co-purify with protein and nucleic acid aggregates^38^. When EVs purified by the two different methods were compared for their protein biomarker signals we found a strong differentiation between cases and controls for two key biomarkers (CA19-9 and CA 125) from the ACE-isolated EVs, but not for the UC-isolated EVs (Fig. 3c, d). A summary of the measurements for the EVs from both isolation techniques is shown in Supplementary Data 8. These results suggest that the ACE EV isolation can be a suitable tool for the purification of EVs directly from plasma and may thus provide a relevant avenue for proteomic analysis. Furthermore, EV isolation using the Verita™ platform is more efficient, the entire process takes about 2 h since no added pre- or post-processing steps are required, it does not rely on immunoaffinities, and it involves less of the sample handling which can damage the EVs^39^. Most importantly, unlike UC, ACE isolation of EVs has the potential to be integrated into high-throughput, automated systems.

Our case-control study involved measurements of the levels of 42 EV-associated protein biomarkers for both the study cohort cancer cases (47 pancreatic, 44 ovarian, 48 bladder) and the controls (184 controls) via a multiplex immunoassay, and an individual assessment of each protein level was performed (protein measurements are shown in Supplementary Data 2 and heatmaps of the normalized protein levels are shown in Fig. S5). In addition. levels of the unpurified, total circulating plasma proteins (“free proteins”) were measured from the same study cohorts (Supplementary Data 2; Fig. S5). To identify the EV-associated protein biomarkers with the largest differentiation potential, a process was employed to select the biomarkers using recursive feature elimination (RFE) with cross-validation^30,34^. The use of repeated cross-validation worked best within the limitations of the sample size for this pilot study (*N* = 323). One hundred repetitions of fivefold cross validation were performed (Fig. 2), and across all repetitions, the RFE algorithm used stepwise backwards selection to arrive at the optimal number of biomarkers that maximized the partial AUC (pAUC)^31^. By optimizing the p(AUC) between specificities of 0.75 and 1.00 we aimed to tailor the biomarker selection towards the reduction of false-positive occurrences (a control mistakenly called as cancer), since this is critical for MCED-type approaches in order to reduce the costs associated with false-positive testing^8^. This strategy resulted in the selection of 13 EV protein markers. After the biomarkers were selected, our cohort was separated at random into a training set (67% of the samples) and a held-out set (33% of the samples) stratified by cancer type (pancreatic, ovarian, and bladder) to estimate the respective coefficients for each biomarker in the logistic regression model exploring the potential for detection of cancer at early stages (Fig. 2). The individual logistic regression coefficients were estimated using the training set, while the performance was evaluated in the held-out test set. Box plots comparing cases and controls for the 13 selected biomarkers are shown in Fig. S6, their coefficient and importance score are shown in Supplementary Data 7, and their Pearson correlation coefficients are shown in Supplementary Data 9 and Fig. S7.

**Fig. 4:**
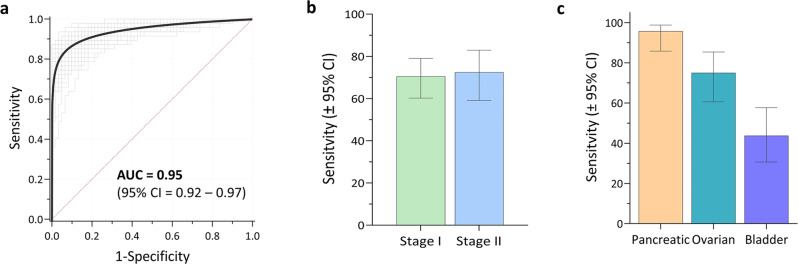
Overall performance for detecting the presence of early cancer using an EV protein-based logistic classifier. **a** ROC curves from comparison of the cancer cases (*N* = 139) to the controls (*N* = 184) on the held-out test sets; black line represents the averaged curve of 100 independently resampled held-out test sets (gray lines). AUC area under the ROC curve. **b** Sensitivities at >99% specificity for detecting either stage I or stage II pancreatic, ovarian, and bladder cancers combined. *N* for stage I cancers is 88 subjects and the *N* for stage II cancers is 51 subjects. **c** Sensitivity at >99% specificity for detecting either stages I and II pancreatic (*N* = 47), ovarian (*N* = 44), or bladder (*N* = 48) cancer. Error bars in both panels (**b**), (**c**) represent the two-sided 95% Wilson confidence intervals.

This performance evaluation was strengthened by employing the widely-used statistical process of resampling which better represents how a larger dataset will perform^30,32,33,40^. By resampling, we could evaluate if the initial random partition created an unrealistic model due to a rare distribution of subjects in that initial partition. We randomly resampled 100 training and test sets (2/3 and 1/3 of the subjects, respectively) from the overall data and generated 100 individual logistic fits for the training portion; from these fits we generated individual ROC curves for the test sets (Fig. 4a). Likewise, each time a subject is featured in the held-out test set, a fit for their logistic model is produced and subsequently averaged among all the times that specific subject was used in a test set (Supplementary Data 5) and from these average fits the overall performance of the model can be assessed. The performance for each of the 100 randomly partitioned test sets can be assessed individually which, when an average threshold for the target specificity of >99% is computed, permits determination of the overall average sensitivity and confidence intervals.

When the overall cancer case cohort was compared with the control individuals using the EV protein biomarker test, the average AUC was found to be 0.95 (95% CI: 0.92–0.97) as shown in Fig. 4a, with an average sensitivity of 71.2% (95% CI: 63.2–78.1) at a specificity of 99.5% (95% CI: 97.0–99.9), as shown in Table 1. For the average of the 100 test sets, the AUC for the exo-proteins was found to be larger than that of the equivalent plasma free-proteins (Fig. S8), at 0.95 vs. 0.87, respectively. When considered across all the three cancers studied, our EV protein test demonstrated sensitivities of 70.5% (95% CI: 60.2–79.0) and 72.5% (95% CI: 59.1–82.9) for stage I and II patients, respectively **(**Fig. 4b and Table 1**)**. Furthermore, we analyzed the sensitivity at >99% specificity for each individual cancer, finding values of 43.8% (95% CI: 30.7–57.7) for bladder cancer, 75.0% (95% CI: 60.6–85.4) for ovarian cancer and 95.7% (95% CI: 85.8–98.8) for pancreatic cancer (Fig. 4c). These results suggest that EV proteins have the potential for detecting early-stage cancers at screening-relevant sensitivities.

**Table 1:** Performance of logistic classifier using EV proteins.

Category	# Subjects	Specificity (%, 95% CI)^a^	Sensitivity (%, 95% CI)^a^
Controls	184	99.5 (97.0–99.9)	
All cancer cases	139		71.2 (63.2–78.1)
Stage I	88		70.5 (60.2–79.0)
Stage II	51		72.5 (59.1–82.9)
Pancreatic cancer	47		95.7 (85.8–98.8)
Ovarian cancer	44		75.0 (58.9–85.4)
Bladder cancer	48		43.8 (30.7–57.7)

^a^Two-sided 95% Wilson confidence intervals.

The 13 EV protein biomarkers identified here span a wide range of biological functions that may represent pivotal points in cancer development. Neuropilin-1 and CA15-3 mediate aberrant growth factor signaling in early malignancies^41,42^. CA 19-9, MPO, and TIMP-1, known cancer drivers, were previously utilized in another multi-cancer test^14^. Neuropilin-1 and sE-selectin are known drivers of angiogenesis processes^43,44^ while exosomal Cathepsin-D, MIA, IGFBP3, sFas, and Ferritin have been shown to impact tumor progression^19,21,45–48^. sFAS has been shown to promote cancer stem cell survival^49^, and bHCG may regulate epithelial to mesenchymal transition events in ovarian cancer cell progression^50^. Several of the proteins have previously been shown to be present in EVs^51–55^. Total serum CA-125 is approved for use in monitoring treatment response and recurrence for ovarian cancer, but it is not recommended to be used as a screening marker^56^. Similarly, total serum CA19-9 is FDA-approved for pancreatic cancer treatment and recurrence monitoring, but importantly, not for screening since on its own CA19-9 may be elevated in several benign conditions^57^.

**Fig. 5:**
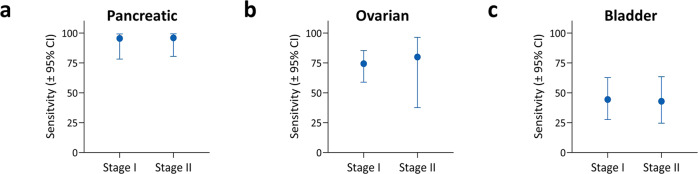
Sensitivities by stage for simultaneous detection of three cancer types using EV protein biomarkers. **a** Sensitivity for detecting either stage I (*N* = 22) or stage II (*N* = 25) pancreatic cancer. **b** Sensitivity for detecting either stage I (*N* = 39) or stage II (*N* = 5) ovarian cancer. **c** Sensitivity for detecting either stage I (*N* = 27) or stage II (*N* = 21) bladder cancer. All sensitivities represent values at >99% specificity for the held-out test sets. Error bars in all panels represent the two-sided 95% Wilson confidence intervals.

To further understand the potential utility of the EV protein-based test, we evaluated performance at stage for each cancer and compared sensitivities at the 99.5% specificity determined from the overall analysis. With the caveat that our sample size for each cancer type was relatively small, the test demonstrated very high sensitivities in detecting both the 22 stage I (95.5%; CI: 78.2–99.2) and 25 stage II PDAC patients (96.0%, CI: 80.5–99.3) (Fig. 5a), indicating a potential breakthrough for the early detection of this malignancy. Detection of stage I ovarian cancer (*N* = 39) was also at levels with potential clinical impact (74.4%, CI: 58.9–85.4) as shown in Fig. 5b. We further broke down our ovarian cancer cohort into both the lethally aggressive serous adenocarcinoma histology (stage I/II, *N* = 22) and stage IA (*N* = 26), showing sensitivities ranging from 68.2% (CI: 47.3–83.6) to 73.1% (CI: 53.9–86.3 CI) at >99% specificity, respectively. Early detection of either subtype could impact survival rates, as surgery would likely be curative. In bladder cancer, the test was able to detect the 27 stage I patients at 44.4% (CI: 27.6–62.7), and the 21 stage II patients at 42.9% (CI: 24.5–63.5) as shown in Fig. 5c. The lower sensitivities for bladder cancer, compared to the high sensitivities for pancreatic and ovarian cancer, may reflect the limited availability of suitable biomarkers for detecting early-stage bladder cancer in the panels we evaluated. In addition, bladder cancer is known to have high molecular and histologic heterogeneity^58,59^.

Taken as a whole, these results suggest that the EV-based protein biomarker test is not biased toward any sub-cohort within each cancer. While PDAC and ovarian cancer detection require ~99% specificity to be viable for population-level screening, an argument could be made that bladder cancer may benefit from a lower specificity threshold. Treatment for late-stage bladder cancer has a significant impact on quality of life and it is among the most expensive cancers to treat in the US^7^. A test with a higher sensitivity may help reduce burden on both patients and the healthcare system by detecting more positives at an early stage where treatment is simpler and does not require removal of the bladder. In the emerging field of MCED testing, our test is unique because while other tests have the potential to improve the prognosis for later-stage cancer^13^, ours can provide higher sensitivity for detection of early-stage cancer, as exemplified by our ~96% sensitivity for stage I and II PDAC cases.

As with any pilot study, there are limitations to acknowledge. First, while informative for biomarker discovery purposes, our relatively small sample cohort, and the inclusion of 100% early-stage tumors do not reflect realistic cancer population characteristics, and sensitivities may be lower when screening large, asymptomatic populations^5,8^. However, since survival is directly linked to detecting cancer early, we decided to exclusively focus our cohort on stages I and II. Second, both cohorts are ethnically homogenous, with sex ratios that may be skewed in comparison to the general frequency observed in cancer between males and females^5^. Third, our control population consisted of individuals without a history of cancer or known confounding comorbidities (e.g., chronic pancreatitis) that in a true screening setting may yield additional false-positive results. Finally, this pilot study will require independent external validation using larger cohorts of blinded samples to verify the potential utility of this MCED approach.

In summary, we have developed a blood-based EV protein detection test and demonstrated its potential role in MCED. The EV protein biomarker test requires <500 µL of plasma and permits integration into an automated workflow. Using a non-invasive blood-based approach, we selected a panel of 13 EV proteins that along with age, a known cofactor in cancer^60^, allowed detection of stage I and II pancreatic, ovarian, and bladder cancers with high diagnostic potential (AUC = 0.95). Most importantly, we obtained a sensitivity of 71.2% at high specificity (99.5%), a key factor for future screening efforts. This test is the first to effectively utilize EVs in early cancer detection via an AC electrokinetic, lab-on-a-chip, scalable platform. Because the Verita™ platform has multi-omic detection capabilities, the addition of other exo-proteins, exosomal mRNA, and/or circulating DNA biomarkers is possible^23^.

The three cancer types studied herein (pancreatic, ovarian, and bladder) are estimated to account for roughly 88,000 deaths in the US in 2021, representing ~14% of all cancer-related deaths^5^. Larger studies are in progress to evaluate this EV protein analysis platform, with the goal of developing a truly effective MCED test capable of providing meaningful information for population-level screening.

## Supplementary information

Description of Additional Supplementary Files

Supplementary Information

Supplementary Data 1

Supplementary Data 2

Supplementary Data 3

Supplementary Data 4

Supplementary Data 5

Supplementary Data 6

Supplementary Data 7

Supplementary Data 8

Supplementary Data 9

Reporting Summary

## Data Availability

All source data for the figures in the main manuscript are contained in Supplementary Data 2, 5, 6 and 8. Source data for figures in the supplementary information are contained in Supplementary Data 2, 3, 8, and 9. Additional datasets cannot be made public at this time due to potential intellectual property or confidentiality limitations. Requests to access additional datasets beyond those described in the manuscript, pertaining to the development of the Verita™ platform and blood test, will promptly undergo an internal review to verify whether the request is subject to any intellectual property or confidentiality limitations. All additional released data and materials will be subject to a data transfer agreement and provided. Requests to access the datasets should be directed to the corresponding authors.

## Code availability

The R package caret (Classification And Regression Training, v6.0-90) used in this study to estimate the model, via recursive feature elimination with cross-validation, is available at https://github.com/topepo/caret. The recursive feature algorithm used in the caret package is described in further detail at https://topepo.github.io/caret/recursive-feature-elimination.html. The code availability is limited to prevent potential misuse in screening people from cancer, to pursue regulatory approval and, to allow for proper IP protection. Interested researchers can contact the corresponding authors for questions on its status and access.
