# Supplementary material for: Early-stage multi-cancer detection using an extracellular vesicle protein-based blood test
Source: Commun Med (Lond). 2022 Mar 17;2:29. doi: 10.1038/s43856-022-00088-6 (PMC9053211; doi:10.1038/s43856-022-00088-6)
Supplement: Supplementary file 1 — Description of Additional Supplementary Files [file 43856_2022_88_MOESM1_ESM.pdf]

Description of Additional Supplementary Files

File Name: Supplementary Data 1

Description: Overview of the subjects included in the cohort with summarized information on sample type, staging, histology, sex, age, and BMI.

File Name: Supplementary Data 2

Description: Detailed information in a per subject basis for sample type, cohort, sex, age, stage, TNM status, histology, and protein biomarker readings.

File Name: Supplementary Data 3

Description: Information in a per subject basis of the nanoparticle tracking analysis results

File Name: Supplementary Data 4

Description: Information of the biomarker's abbreviations, full names, limit of detection values and units of measure.

File Name: Supplementary Data 5

Description: Average subject fit from the logistic classifier model and the number of times the subject was featured on the held-out test sets.

File Name: Supplementary Data 6

Description: AUC and threshold values in the held-out test sets for the logistic classifier model built from the training set splits.

File Name: Supplementary Data 7

Description: Logistic regression model coefficients and importance scores for each of the biomarkers used in the logistic classifier.

File Name: Supplementary Data 8

Description: Subject information, plasma protein quantification, nanoparticle tracking analysis and, key biomarker levels for the cohort used in comparison with differential ultracentrifugation.

File Name: Supplementary Data 9

Description: Pearson correlation coefficients for the biomarkers used in the logistic classifier
