# Supplementary material for: Early-stage multi-cancer detection using an extracellular vesicle protein-based blood test
Source: Commun Med (Lond). 2022 Mar 17;2:29. doi: 10.1038/s43856-022-00088-6 (PMC9053211; doi:10.1038/s43856-022-00088-6)
Supplement: Supplementary file 2 — Supplementary Information [file 43856_2022_88_MOESM2_ESM.pdf]

SUPPLEMENTARY INFORMATION

Early-Stage Multi-Cancer Detection Using an Extracellular Vesicle Protein-Based Blood Test

Juan Pablo Hinestrosa[1], Razelle Kurzrock[2,3], Jean M. Lewis[1], Nicholas J. Schork[4,5,6*], Gregor Schroeder[1], Ashish M. Kamat[7], Andrew M. Lowy[2], Ramez Eskander[2], Orlando Perrera[1], David Searson[1], Kiarash Rastegar[1], Jake R. Hughes[1], Victor Ortiz[1], Iryna Clark[1], Heath I. Balcer[1], Larry Arakelyan[1], Robert Turner[1], Paul R. Billings[1], Mark Adler[8], Scott M. Lippman[2*], Rajaram Krishnan[1]*

1. *Biological Dynamics, Inc., San Diego, CA, USA*
2. *University of California San Diego, Moores Cancer Center, San Diego, CA, USA*
3. *WIN Consortium for Personalized Cancer Therapy, Villejuif, France*
4. *The Translational Genomics Research Institute (TGen), Phoenix, AZ, USA*
5. *City of Hope National Medical Center, Duarte, CA, USA*
6. *University of California San Diego, Departments of Family Medicine and Public Health, San Diego, CA, USA*
7. *University of Texas MD Anderson Cancer Center, Department of Urology, Houston, TX, USA*
8. *San Diego Cancer Research Institute, San Diego, CA, USA*

*Corresponding Authors: Nicholas J. Schork (NJS) (nschork@tgen.org), Scott M. Lippman (slippman@health.ucsd.edu), Rajaram Krishnan (RK2) (raj@biologicaldynamics.com)

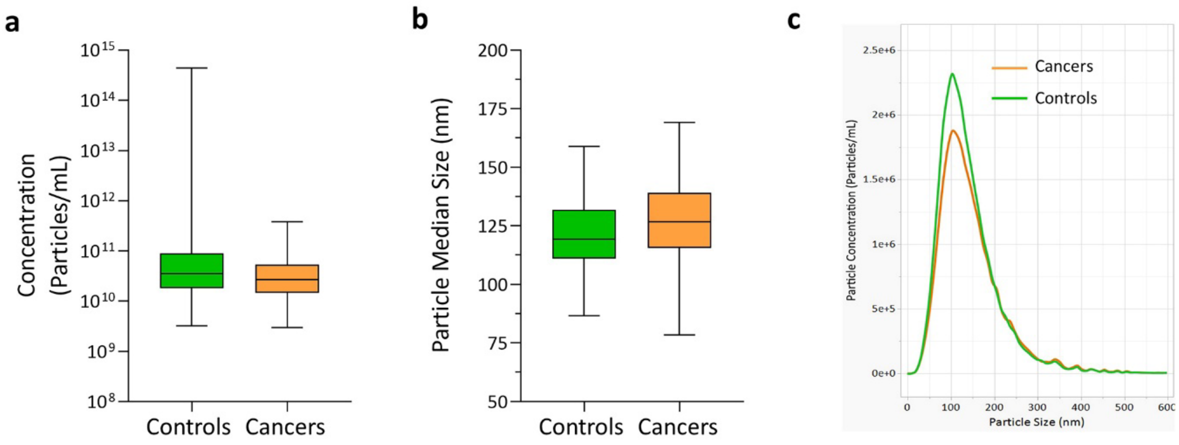

Figure S1: Comparison of NTA results for control and cancer cases. a. Particle concentration for Verita™-purified EVs, shown for cancer cases or controls. **b,** Particle median size for Verita™-purified EV particles, shown for cancer cases or controls. In panels **a** and **b**, each box represents the min to max (lower and upper whiskers) and the 25%, 50% and 75% percentiles (box lines). **c,** Overall EV particle size distributions for cancers and controls. In all panels, the N for controls is 162 subjects and the N for cancers is 136 subjects.

a

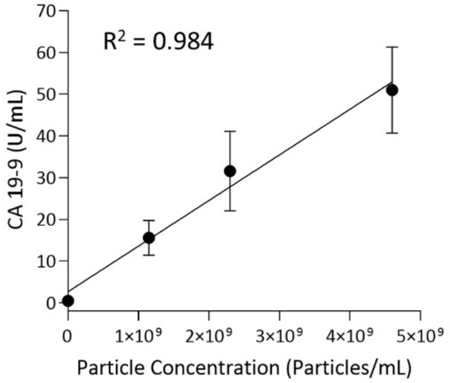

H1975 EV Spike (Particles/mL)	CA 19-9 (U/mL)	
	Mean	SD
**4.6 x 10⁹**	51.0	10.3
**2.3 x 10⁹**	31.6	9.5
**1.15 x 10⁹**	15.6	4.2
**No Spike (plasma only)**	0.5	0.2

b

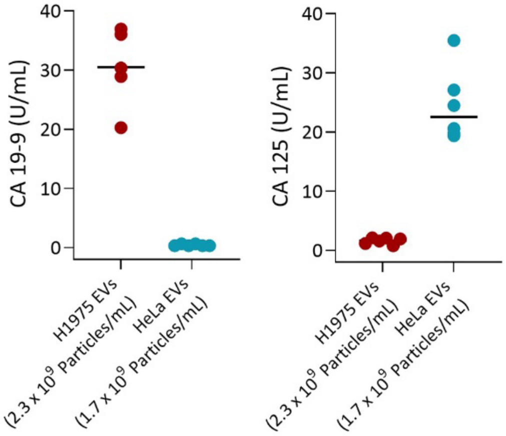

EV Spike	CA 19-9 (U/mL)		CA 125 (U/mL)	
	Mean	SD	Mean	SD
**H1975**	30.5	6.0	1.6	0.5
**HeLa**	0.4	0.1	24.5	6.2

Figure S2: Performance of assay using purified cell culture EVs spiked into K_2EDTA plasma at known particle concentrations. a. The levels of CA 19-9 measured in H1975 EVs at three different particle concentrations (N = 5 replicates for each concentration) shows a linear response with EV input. The K_2EDTA plasma with no EV spike showed negligible concentration of this marker. The error bars represent the standard deviation. **b.** Detection of CA19-9 is positive in H1975 cell EVs (N = 5 replicates), but not in HeLa cell EVs (N = 5 replicates) spiked into K_2EDTA plasma; detection of CA125 is positive for spiked HeLa cell EVs (N = 5 replicates), but very low for spiked H1975 cell EVs (N = 5 replicates).

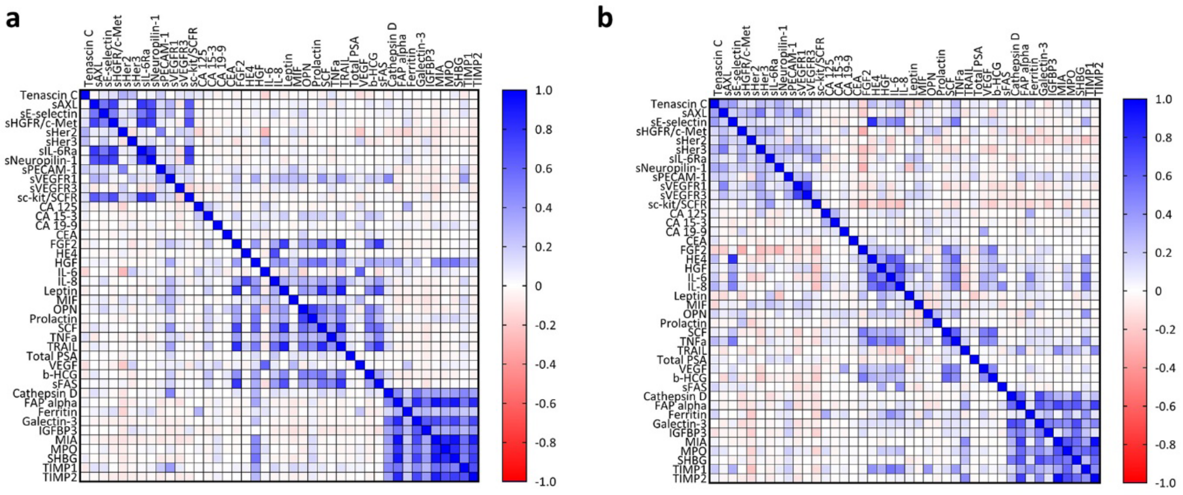

Figure S3: Pearson Correlation Coefficients for Measured Protein Levels. a. EV-associated Proteins. **b.** Free Proteins.

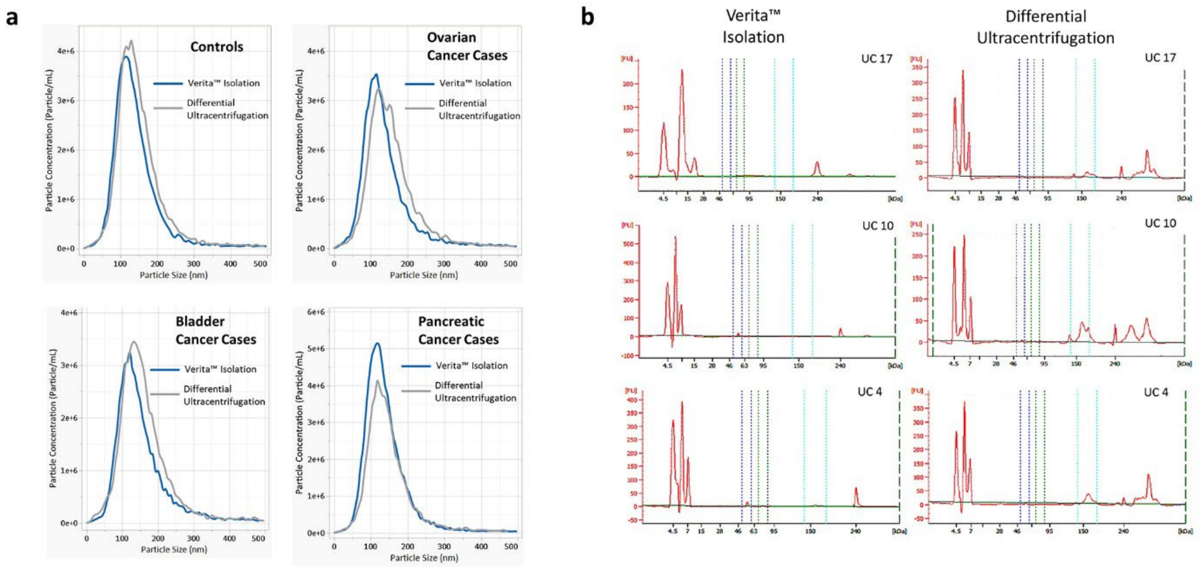

Figure S4: Characterization of EVs isolated using either Verita™ or Differential Ultracentrifugation. a Particle size distribution shown for controls (N = 11) and for ovarian (N = 4), bladder (N = 4), and pancreatic (N = 6) cancer cases. Blue lines, Verita-isolated EVs; grey lines, differential ultracentrifugation-isolated EVs. **b.** Protein bioanalyzer electropherograms for selected samples. The blue dashed lines show the protein size range for Albumin (50 to 60kDa), the green dashed lines show the same range for Fibrinogen (70-85kDa) and the cyan dash lines show the range for IgG (140-180kDa).

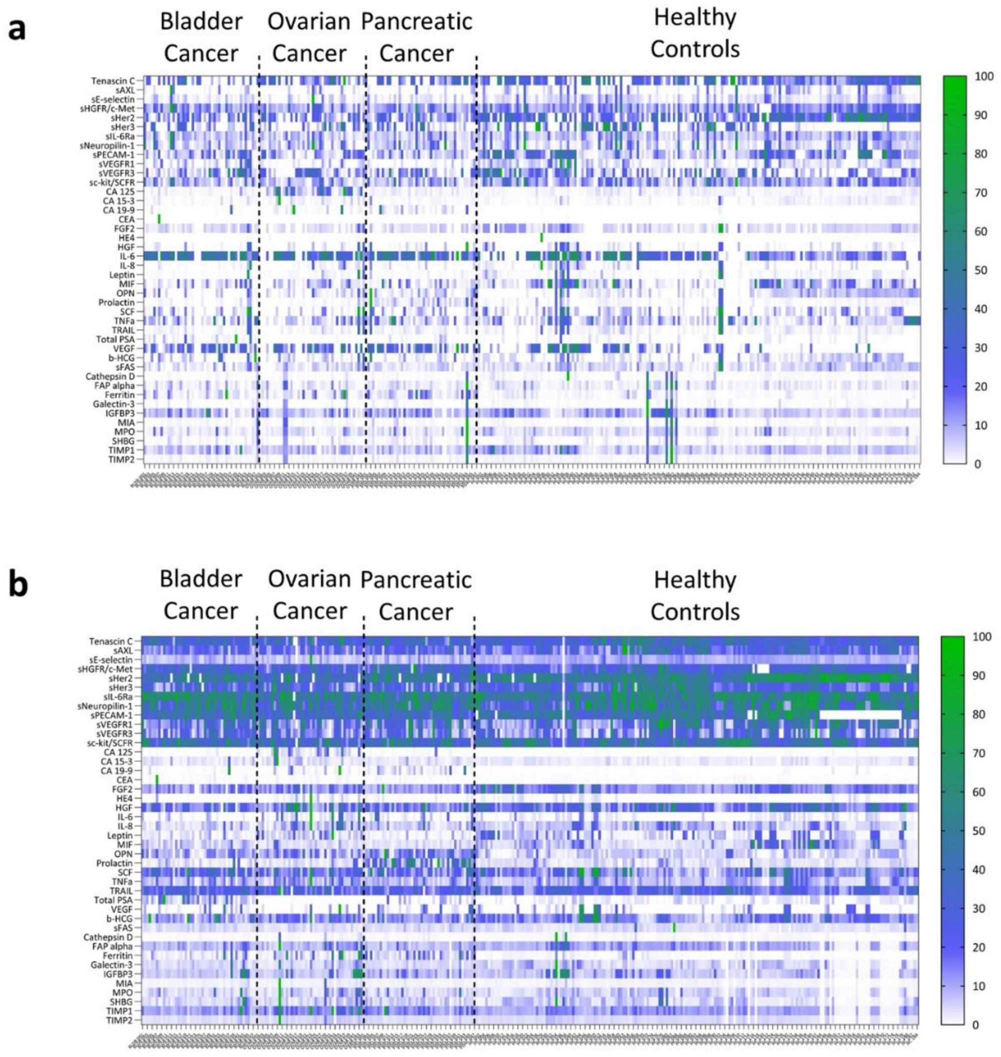

Fig. S5: Heatmap of normalized concentration values for analyzed proteins for cancer and control cases. a. EV-associated proteins. **b.** Free Proteins. Normalization is across the entire cohort for each biomarker; each column represents a subject in the study. In both panels the N values for bladder, ovarian, pancreatic, and healthy controls is 48, 44, 47 and 184 subjects respectively.

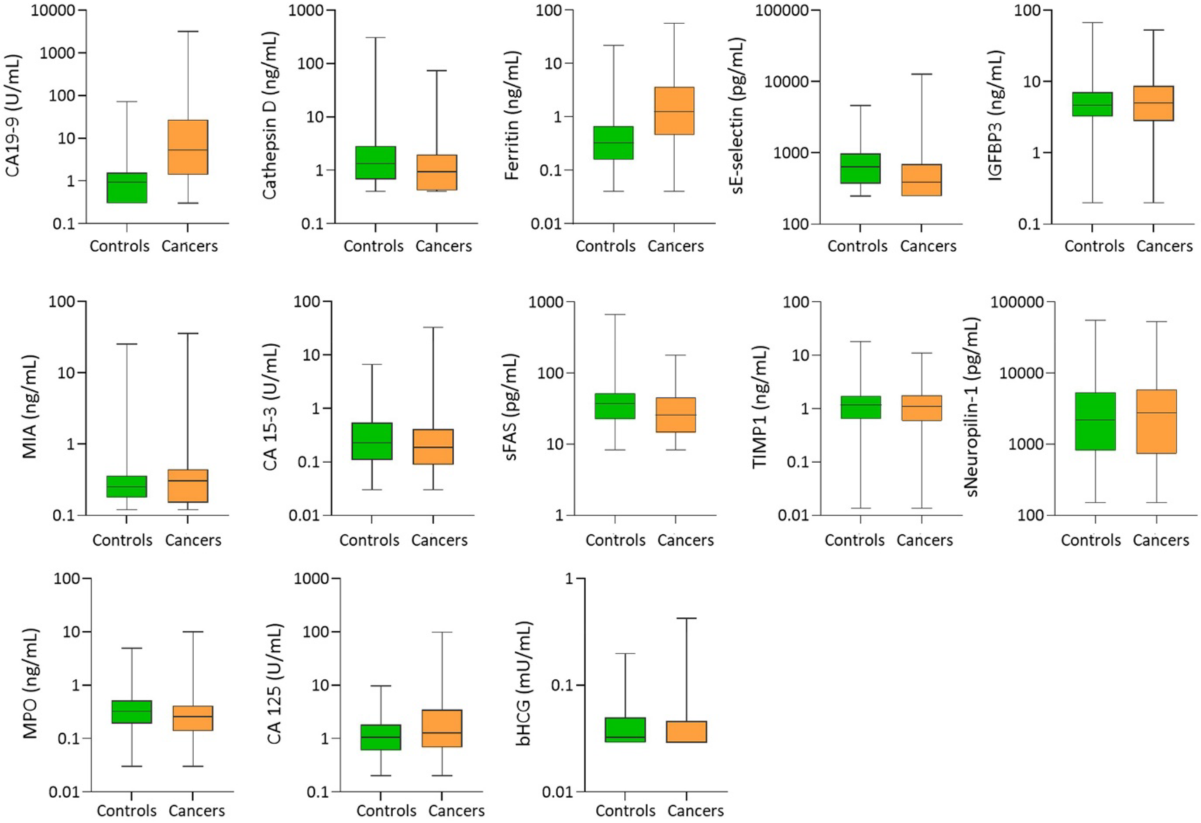

CA19-9 (U/mL)
10000
1000
100
10
1
0.1
Controls
Cancers
Cathepsin D (ng/mL)
1000
100
10
1
0.1
Controls
Cancers
Ferritin (ng/mL)
100
10
1
0.1
0.01
Controls
Cancers
sE-selectin (pg/mL)
100000
10000
1000
100
Controls
Cancers
IGFBP3 (ng/mL)
100
10
1
0.1
Controls
Cancers
MIA (ng/mL)
100
10
1
0.1
Controls
Cancers
CA 15-3 (U/mL)
100
10
1
0.1
0.01
Controls
Cancers
sFAS (pg/mL)
1000
100
10
1
Controls
Cancers
TIMP1 (ng/mL)
100
10
1
0.1
0.01
Controls
Cancers
sNeuropilin-1 (pg/mL)
100000
10000
1000
100
Controls
Cancers
MPO (ng/mL)
100
10
1
0.1
0.01
Controls
Cancers
CA 125 (U/mL)
1000
100
10
1
0.1
Controls
Cancers
bHCG (mU/mL)
1
0.1
0.01
Controls
Cancers

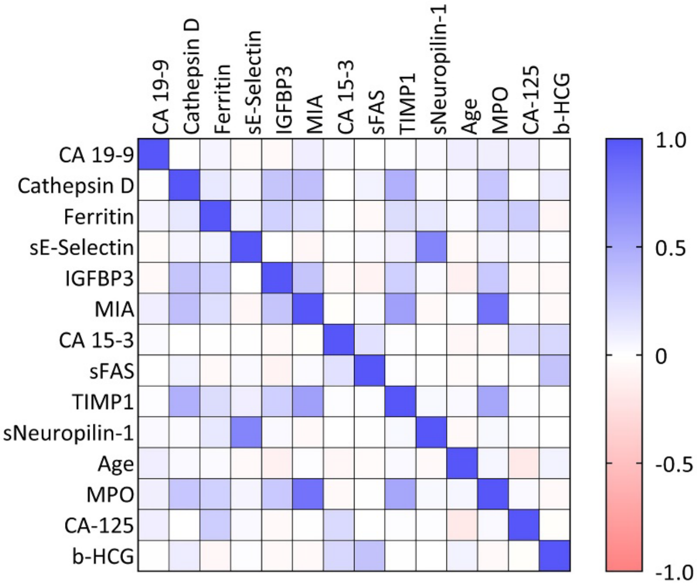

Figure S7: Pearson Correlation Coefficients for Biomarkers Selected in the Logistic Classifier Model. As shown in key, values above 0 represent positive correlations, with values below 0 representing negative correlations between each pair markers.

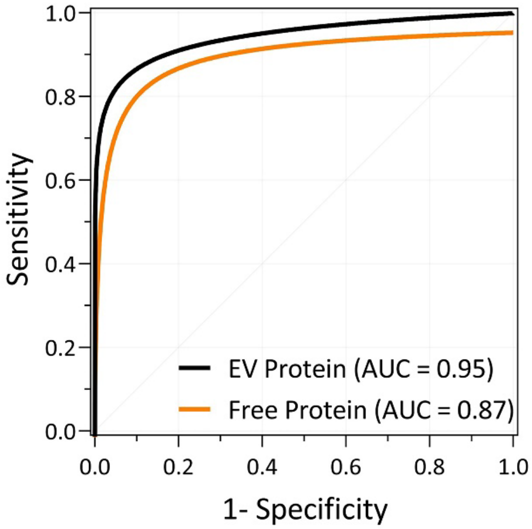

Figure S8: ROC Comparison between EV-associated Proteins and Free Proteins. ROC curves were generated using the protein concentrations derived from the exo-proteins (black line) or free proteins (orange line) using the biomarkers selected in the logistic classifier model. The same cohort (N = 184 for controls, N = 139 for cases) was employed in the calculation.
