# Supplementary material for: Early-stage multi-cancer detection using an extracellular vesicle protein-based blood test
Source: Commun Med (Lond). 2022 Mar 17;2:29. doi: 10.1038/s43856-022-00088-6 (PMC9053211; doi:10.1038/s43856-022-00088-6)
Supplement: Supplementary file 12 — Reporting Summary [file 43856_2022_88_MOESM12_ESM.pdf]

nature research

Corresponding author(s): Rajaram Krishnan

Last updated by author(s): 2022/02/01

Reporting Summary

Nature Research wishes to improve the reproducibility of the work that we publish. This form provides structure for consistency and transparency in reporting. For further information on Nature Research policies, see our Editorial Policies and the Editorial Policy Checklist.

Statistics

For all statistical analyses, confirm that the following items are present in the figure legend, table legend, main text, or Methods section.

n/a	Confirmed	
☐	☒	The exact sample size (*n*) for each experimental group/condition, given as a discrete number and unit of measurement
☐	☒	A statement on whether measurements were taken from distinct samples or whether the same sample was measured repeatedly
☐	☒	The statistical test(s) used AND whether they are one- or two-sided<br>*Only common tests should be described solely by name; describe more complex techniques in the Methods section.*
☐	☒	A description of all covariates tested
☐	☒	A description of any assumptions or corrections, such as tests of normality and adjustment for multiple comparisons
☐	☒	A full description of the statistical parameters including central tendency (e.g. means) or other basic estimates (e.g. regression coefficient) AND variation (e.g. standard deviation) or associated estimates of uncertainty (e.g. confidence intervals)
☒	☐	For null hypothesis testing, the test statistic (e.g. *F*, *t*, *r*) with confidence intervals, effect sizes, degrees of freedom and *P* value noted<br>*Give P values as exact values whenever suitable.*
☒	☐	For Bayesian analysis, information on the choice of priors and Markov chain Monte Carlo settings
☒	☐	For hierarchical and complex designs, identification of the appropriate level for tests and full reporting of outcomes
☐	☒	Estimates of effect sizes (e.g. Cohen's *d*, Pearson's *r*), indicating how they were calculated

Our web collection on statistics for biologists contains articles on many of the points above.

Software and code

Policy information about availability of computer code

Data collection	Data was collected using a commercial immunoassay kit for the protein expression of both exo- and free-proteins
Data analysis	Data analysis was performed mainly in the software package R (v 3.6.0) using custom code. In addition, other statistical analysis and plotting were performed in the software packages JMP Pro (v 16.1.0) and GraphPad Prism (v 9.1.2)

For manuscripts utilizing custom algorithms or software that are central to the research but not yet described in published literature, software must be made available to editors and reviewers. We strongly encourage code deposition in a community repository (e.g. GitHub). See the Nature Research guidelines for submitting code & software for further information.

Data

Policy information about availability of data

All manuscripts must include a data availability statement. This statement should provide the following information, where applicable:

- Accession codes, unique identifiers, or web links for publicly available datasets
- A list of figures that have associated raw data
- A description of any restrictions on data availability

Source data for the figures in the main manuscript can be accessed from the Supplementary Data 2, 5, 6 and 8. Additional data requests will promptly undergo an internal review to verify whether the request is subject to any intellectual property or confidentiality obligations. Any released data and materials will be subject to a data transfer agreement. Requests to access the datasets should be directed to the corresponding authors.

Field-specific reporting

Please select the one below that is the best fit for your research. If you are not sure, read the appropriate sections before making your selection.

☒ Life sciences ☐ Behavioural & social sciences ☐ Ecological, evolutionary & environmental sciences

For a reference copy of the document with all sections, see nature.com/documents/nr-reporting-summary-flat.pdf

Life sciences study design

All studies must disclose on these points even when the disclosure is negative.

Sample size	In this pilot study we tested 139 cancer patients (Pancreatic, Ovarian and Bladder) in comparison with 184 control. The goal was to determine the best set of markers and parameters for differentiation
Data exclusions	The data exclusions are described on the materials and methods section of the manuscript.
Replication	All measurements were done in duplicates with a selected set done in triplicates or larger Ns
Randomization	The randomization of the data occurred during the analysis portion for the creation on the test where 100 randomly selected partitions into training and test sets were performed. The training partition is used to generate the coefficients for each biomarker in the logistic regression and then the performance was evaluated in the held-out test set.
Blinding	Blinding does not apply

Reporting for specific materials, systems and methods

We require information from authors about some types of materials, experimental systems and methods used in many studies. Here, indicate whether each material, system or method listed is relevant to your study. If you are not sure if a list item applies to your research, read the appropriate section before selecting a response.

Materials & experimental systems

n/a	Involved in the study	
☐	☒	Antibodies
☐	☒	Eukaryotic cell lines
☒	☐	Palaeontology and archaeology
☒	☐	Animals and other organisms
☐	☒	Human research participants
☐	☒	Clinical data
☒	☐	Dual use research of concern

Methods

n/a	Involved in the study	
☒	☐	ChIP-seq
☒	☐	Flow cytometry
☒	☐	MRI-based neuroimaging

Antibodies

Antibodies used	Bead-based immunoassay kits (Human Circulating Biomarker Magnetic Bead Panel 1 (Cat # HCCBP1MAG-58K), Human Angiogenesis Magnetic Bead Panel 2 (Cat # HANG2MAG-12K), and Human Circulating Cancer Biomarker Panel 3 (Cat # HCCBP3MAG-58K)) were procured from a commercial source (Millipore Sigma, Burlington, MA).
Validation	Extracted EV/exosomes samples and plasma from the same patients were tested for concentration of target proteins on the MAGPIX system (Luminex Corp, Austin, TX) according to manufacturer's protocols. Belysa software v. 3.0 (EMD Millipore) was used to determine final protein concentrations from the calibration curves.

Eukaryotic cell lines

Policy information about cell lines

Cell line source(s)	ATCC. For H1975 : ATCC CRL-5908 and for HeLa: ATCC CRM-CCL-2
Authentication	yes, STR and phenotyping, by ATCC
Mycoplasma contamination	Not to our knowledge
Commonly misidentified lines (See ICLAC register)	Not to our knowledge

Human research participants

Policy information about studies involving human research participants

Population characteristics	A total of 323 subjects were included in the study, including 139 subjects ('Cancer case patient cohort') who were diagnosed with one of the three cancers between January 2014 and September 2020. In the cancer case cohort, whole venous blood specimens were collected shortly before biopsy (median -1 day, mean -2.7 days), and prior to surgical intervention, radiation therapy, or cancer-related systemic therapy. Median age was 60 years [Min – Max 21-76] in the cancer case cohort (N=139, 56 males, 83 females) and 57 years [Min – Max 40-71] in the control cohort (N=184, 82 males and 82 females).
Recruitment	Samples were obtained from a commercial biobank
Ethics oversight	All specimens for this study were obtained from a commercial biorepository (ProteoGenex, Culver City, CA, USA). All relevant ethical regulations were followed, and informed consent was obtained prior to sample collection. The protocol was approved by the ethics committee at the N. N. Blokhin National Medical Research Center of Oncology.

Note that full information on the approval of the study protocol must also be provided in the manuscript.

Clinical data

Policy information about clinical studies

All manuscripts should comply with the ICMJE guidelines for publication of clinical research and a completed CONSORT checklist must be included with all submissions.

Clinical trial registration	*Provide the trial registration number from ClinicalTrials.gov or an equivalent agency.*
Study protocol	*Note where the full trial protocol can be accessed OR if not available, explain why.*
Data collection	*Describe the settings and locales of data collection, noting the time periods of recruitment and data collection.*
Outcomes	*Describe how you pre-defined primary and secondary outcome measures and how you assessed these measures.*
